# Prostate Cancer Presenting with Parietal Bone Metastasis

**DOI:** 10.1155/2017/1928570

**Published:** 2017-03-09

**Authors:** Brahima Kirakoya, Abdoul Karim Pare, Babagana Mustapha Abubakar, Moussa Kabore

**Affiliations:** ^1^Urology Department, University Hospital Yalgado Ouedraogo, Ouagadougou, Burkina Faso; ^2^Department of Urology, University Hospital Souro Sanou, Bobo Dioulasso, Burkina Faso; ^3^Department of Surgery, Federal Medical Center, PMB 02, Nguru, Yobe State, Nigeria

## Abstract

Bone metastases from prostate cancer are very common. They are usually located on the axial skeleton. However, cranial bone metastases especially to the parietal bone are rare. We report a case of metastatic prostate cancer presenting with left parietal bone metastasis in a patient with no urological symptoms or signs. We should consider prostate cancer in any man above 60 years presenting unusual bone lesions.

## 1. Introduction

Prostate cancer is known to have high metastatic potential especially to the bone. Bone metastases are common and are osteoblastic in more than 95% [1]. They represent nearly 40% of secondary locations at diagnosis in Burkina Faso [2]. Metastases to the skull are rare and account for only 2% of secondary locations as reported in an autopsy series [3]. The base of the skull is the most affected. Clinical presentation depends on the location of the metastatic lesion and cranial nerve involved [4]. We report a case of occult prostate cancer presenting with skull metastatic.

## 2. Case Report

A 66-year-old man who was referred to urology department from neurosurgery department presents with one-year history of left parietal region swelling. The swelling progressively increased in size but there was no history of pain or neurological deficit. There was associated easy fatigability and generalized bone pains. Visual acuity and hearing were normal. No urological symptoms were found. Physical examination revealed swelling on the left parietal region that was hard in consistency, slightly tender, and well demarcated without extending to the contralateral side. The overlying skin and hair appear normal. Digital rectal examination revealed enlarged prostate that was nontender, hard, and nodular. Hemoglobin was 11 g/dl. Renal function was normal. The PSA values were 9202 ng/ml. Prostate biopsy histology revealed adenocarcinoma with Gleason score of 3 + 3 = 6.

**Figure 1:**
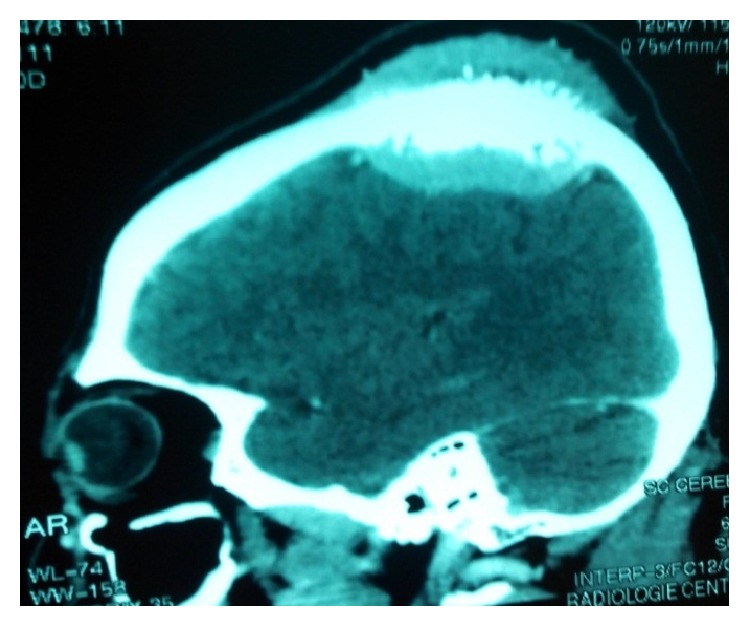
CT of the brain at presentation showing osteoblastic mass on the skull.

**Figure 2:**
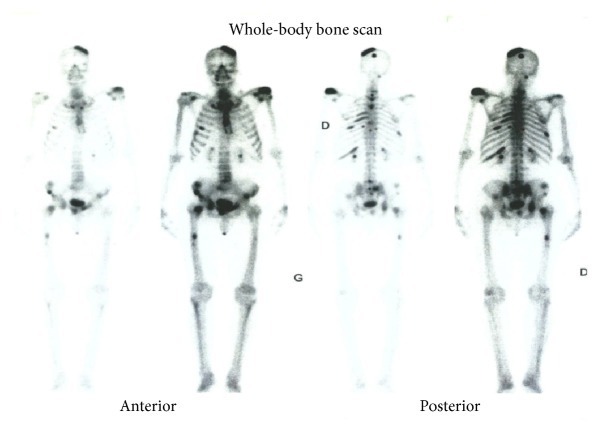
Bone scintigraphy showing metastatic lesions on the left parietal bone, left humeral head, and the spines.

**Figure 3:**
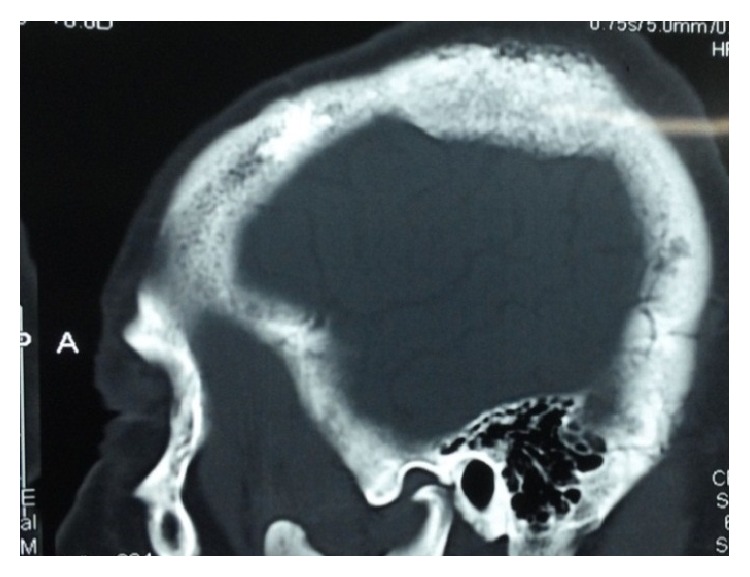
CT of the brain 12 months after androgen deprivation showing decrease in size of the parietal mass and bony exostosis.

Computed tomography (CT) showed a well localized left parietal mass with prominent intra- and extracranial bony exostosis (Figure 1). The whole body bone scan showed areas of high radioactive uptake in the region of the parietal mass, left humeral head, and rib cage (Figure 2). Patient was prepared and had androgen deprivation therapy using Cyproterone at dose of 300 mg per day. More appropriate treatment (LH-RH analogues, abiraterone, or chemotherapy) was above the resources of the patient. He was reviewed at 2nd, 5th, 8th, and 12th month; PSA done at these visits were found to be 5 ng/ml, 1.3 ng/ml, 3 ng/ml, and 20 ng/ml, respectively. The clinical symptoms improved significantly from the 8th month. CT scan performed at 12th month showed decrease in parietal bone swelling and the bony exostosis earlier noted (Figure 3). At 18th month, patient started having progressive body weakness and generalized bone pains. Hemoglobin was then found to be 8 g/dl, serum total PSA was 102 ng/ml, and serum testosterone levels were 0.1 ng/ml. The patient's general condition progressively deteriorated and he died 21 months from commencement of androgen deprivation.

## 3. Discussion

Prostate cancer is the commonest urologic malignancy in both developed and developing countries. Its incidence is increasing due to the improvement in the life expectancy and availability of screening tools such as the PSA. However, there are many patients that present late with advanced disease. At diagnosis, 40.2% of prostate cancers are metastatic in Burkina Faso [2]. Bone metastases are preferentially to the axial skeleton especially the pelvis, lumbar/sacral spine, and rib cage [2, 4]. Cranial metastases are rare and are often discovered at autopsy [5]. However, prostate is the commonest cause of metastatic lesions in the skull [6]. Approximately 12 to 18% of the metastatic lesions to the skull are prostatic in origin [6]. Cranial sites metastases from prostate cancer as well as their relative frequency are well explained by the work of Batson [7]. They do not have a specific clinical presentation, and it all depends on location of the mass. Parietal bone metastases are rare [8]. Swelling caused by bony exostosis may be the only clinical sign. Factors such as age, duration of symptoms, and bone pains may be a pointer to the diagnosis. These features have been reported by some authors to distinguish metastatic lesions from primary lesions in the skull [5]. Our patient has no evidence of urological symptoms; prostatic cancer was suspected because of high index of suspicion. Isolated cranial metastases from prostate cancers are rare; it is usually associated with other bony metastases. Saitoh et al. in an autopsy study found isolated cranial metastases in 6.8% of the cases [3]. The bone scintigraphy done in our case showed multiple hot spot lesions in the skeleton. The very high value of PSA (9202 ng/ml) not only indicates advanced stage but also suggests bone metastasis. According to Wolff et al., PSA levels greater than 100 ng/ml are strongly predictive of bone metastases [9]. In metastatic stage, the aim of treatment is basically palliative in the form of androgen deprivation. Our patient opted for medical castration. The decrease in size of the parietal swelling on CT scan and lower serum PSA levels are indicators of a good biological response of the disease. Rise in PSA despite low testosterone levels (0.1 ng/ml) marks the beginning of Castration Resistance Prostate Cancer (CRPC). Classically, CRPC appears on the average after 24 months of androgen deprivation.

## 4. Conclusion

Patient with prostate cancer, even in advanced stage, may have no urological symptom or sign. Metastatic cranial lesion especially to the parietal bone from prostate cancer is rare. Only high index of suspicion based on age, sex, PSA, and radiological evidence of osteoblastic lesions on CT scan may suggest prostate cancer as the primary site.
